# Over-Expression of CHD4 Is an Independent Biomarker of Poor Prognosis in Patients with Rectal Cancers Receiving Concurrent Chemoradiotherapy

**DOI:** 10.3390/ijms20174087

**Published:** 2019-08-21

**Authors:** Hui-Ching Wang, Chia-Lin Chou, Ching-Chieh Yang, Wei-Lun Huang, Yin-Chou Hsu, Chi-Wen Luo, Tzu-Ju Chen, Chien-Feng Li, Mei-Ren Pan

**Affiliations:** 1 Graduate Institute of Clinical Medicine, College of Medicine, Kaohsiung Medical University, Kaohsiung 807, Taiwan; 2 Division of Hematology and Oncology, Department of Internal Medicine, Kaohsiung Medical University Hospital, Kaohsiung Medical University, Kaohsiung 807, Taiwan; 3 Division of Colon & Rectal Surgery, Department of Surgery, Chi Mei Medical Center, Tainan 710, Taiwan; 4 Institute of Biomedical Sciences, National Sun Yat-sen University, Kaohsiung 804, Taiwan; 5 Department of Radiation Oncology, Chi-Mei Medical Center, Tainan 710, Taiwan; 6 Department of Pharmacy, Chia-Nan University of Pharmacy and Science, Tainan 71745, Taiwan; 7 Department of Radiation Oncology, Kaohsiung Veterans General Hospital, Kaohsiung 813, Taiwan; 8 Department of Emergency Medicine, E-Da Hospital, I-Shou University, Kaohsiung 824, Taiwan; 9 Division of Breast Surgery, Department of Surgery, Kaohsiung Medical University Hospital, Kaohsiung 807, Taiwan; 10 Department of Pathology, Chi Mei Medical Center, Tainan 710, Taiwan; 11 Department of Optometry, Chung Hwa University of Medical Technology, Tainan 717, Taiwan; 12 Department of Medical Research, Chi Mei Medical Center, Tainan 710, Taiwan; 13 National Institute of Cancer Research, National Health Research Institute, Tainan 704, Taiwan; 14 Drug Development and Value Creation Research Center, Kaohsiung Medical University, Kaohsiung 807, Taiwan

**Keywords:** CHD4, CCRT, rectal cancers, radioresistance

## Abstract

Neoadjuvant concurrent chemoradiotherapy (CCRT), followed by radical proctectomy, is the standard treatment for locally advanced rectal cancer. However, a poor response and therapeutic resistance continue to occur despite this treatment. In this study, we analyzed the microarray datasets (GSE68204) of rectal cancer from the Gene Expression Omnibus database, and identified CHD4 as one of the most significantly up-regulated genes among all subunits of the nucleosome remodeling and histone deacetylation (NuRD) complex, in non-responders to CCRT, among locally advanced rectal cancer (LARC) patients. We confirmed the predictive and prognostic significance of CHD4 expression in CCRT treatment, and its correlation with other clinicopathological features, such as tumor regression grade (TRG), therapeutic response, and patient survival. This was carried out by immunohistochemical studies on endoscopic biopsy tissues from 172 rectal cancer patients, receiving neoadjuvant concurrent chemoradiotherapy (CCRT). A high expression of CHD4 was significantly associated with pre-treatment tumor status (*p* < 0.001) and lymph node metastasis (*p* < 0.001), post-treatment tumor status (*p* < 0.001), and lymph node metastasis (*p* < 0.001), vascular invasion (*p* = 0.042), and tumor regression grade (*p* = 0.001). A high expression of CHD4 could also predict poor disease-specific survival and metastasis-free survival (log-rank test, *p* = 0.0373 and *p* < 0.0001, respectively). In multivariate Cox proportional-hazards regression analysis, CHD4 overexpression was an independent factor of poor prognosis for metastasis-free survival (HR, 4.575; 95% CI, 1.717–12.192; *p* = 0.002). By in vitro studies, based on cell line models, we also demonstrated that, the overexpression of CHD4 induced radio-resistance in microsatellite instability-high (MSI-H) colorectal cells (CRCs). On the contrary, the knockdown of CHD4 enhanced radiosensitivity in microsatellite stable (MSS) CRCs. Altogether, we have identified CHD4 as an important regulator of radio-resistance in both MSI-H and MSS CRC cell lines.

## 1. Introduction

The incidence of colorectal cancer (CRC) has been progressively increasing in recent decades; currently, CRC is the third most common form of malignancy in the United States [1]. Rectal cancer accounts for approximately 30% of CRC, and is presented with inferior clinical outcomes [2]. For early stages of rectal cancer, surgical resection remains the main strategy to circumvent the disease. However, for locally advanced rectal cancer, neoadjuvant concurrent chemoradiotherapy (CCRT) is the standard protocol for better survival of patients and functional preservation of the sphincter [2,3,4,5]. Despite the development of these multiple therapeutic approaches, there is a high rate of recurrence and metastasis to distant sites in rectal cancers [6,7]. Therefore, it is important to identify potential biomarkers, that can distinguish poor responders to CCRT from the good responders, among rectal cancer patients. Recent research has revealed that microsatellite instability (MSI) status in rectal cancer can influence tumor classification, therapeutic implications, and disease prognosis [6,7]. Histological studies, combined with transcriptomic data and protein-protein interaction studies are, therefore, required to identify potential biomarkers and categorize patients, based on responses to treatment, and provide information on the risk of recurrence, as well as guide physicians towards combination therapies for poor responders.

Current therapeutic strategies for rectal cancer, include alkylating agents (such as platinum compounds like cisplatin), antimetabolites, and radiotherapy, which mainly induces DNA damage via different modes of action [8,9,10]. Adaption and survival, following treatment with these DNA-damaging agents, also results in increased risk of cancers [11]. The ability of the cellular repair machinery to resist DNA damage determines the development of malignancy and cancer response, following chemotherapy and radiotherapy [12]. Somatic and germline mutations are reported to occur frequently in genes, related to the DNA mismatch repair (MMR) pathway, and are associated with tumorigenesis in CRC patients [13]. The analysis of inheritable defects in the DNA-repair machinery of rectal cancer patients revealed high rates of mutations in homologous recombination (HR) pathway genes, such as *ATM* and *MRE11*. These findings have renewed the interest in targeted interventions with poly-ADP ribose polymerase inhibitors [14]. Thus, it is necessary to understand the interplay between genes, that are related to the DNA repair pathway and rectal cancers, in order to better understand treatment responses and identify potential targets for treatment.

The nucleosome remodeling and histone deacetylation (NuRD) complex is a multi-subunit complex, composed of histone deacetylases 1 and 2 (HDAC1/2), SWI/SNF-type ATPase, helicase-like ATPases chromodomain helicase DNA-binding protein 3 and 4 (CHD3/4), metastasis-associated proteins 1, 2, and 3 (MTA1/2/3), histone chaperone retinoblastoma-binding proteins 4 and 7 (RBBP4/7), zinc-finger proteins GATAD2A/B, cyclin dependent kinase 2 associated protein 1 (Cdk2ap1), and methyl-DNA binding proteins 2 and 3 (MBD2/3) [15,16]. The NuRD complex possesses both, nucleosome remodeling and histone deacetylase activities, which regulate DNA repair and the transcription of a number of target genes. In response to DNA damage, the NuRD complex can be recruited to the damage site and help in DNA repair by multiple mechanisms. Chromodomain helicase DNA-binding protein 4 (CHD4) is a vital component of the NuRD complex, which is associated with both activation and repression of gene transcription regulating cancer, DNA double-strand break repair, stem cell renewal, and cell cycle [17,18,19,20]. CHD4 regulates DNA-damage responses, through its N-terminal region in a poly(ADP-ribose) polymerase-dependent manner [17,21], and maintains the genomic integrity and stability by regulating HR repair. Loss of CHD4 promotes resistance to DNA-damaging agents such as cisplatin [22,23]. Regarding gene regulation, previous study indicated that CHD4 cooperates with DNA methyltransferases (DNMTs) in the silencing of many tumor suppressor genes (TSGs), including *MLH1, SFRP1, SFRP2, SFRP4, TIMP2,* and *TIMP3* to drive the Wnt pathway in CRC cells [24]. This suggests that CHD4 may affect cancer behavior and treatment responses to various cancers. However, there are no reports on the correlation between CHD4 expression and therapeutic responses to CCRT in rectal cancers, with respect to MSI status.

Given the role of CHD4 in the radiotherapy-resistant phenotype, we sought to address the clinical relevance of CHD4 in human cancers. In the present study, tissue samples and bioinformatics were used to assess the role of CHD4 in radiotherapy response. In the in vivo-based approach, the levels of CHD4 protein expression were evaluated in 172 pairs of cancer tissue samples, and adjacent normal mucosa from patients with rectal cancer, who are receiving neo-adjuvant CCRT, followed by surgery. The role of CHD4 was elucidated by analyzing the relationships between clinical and pathological features, including tumor response after CCRT. We also elucidated the prognostic significance of CHD4 expression in the survival of rectal cancer patients. For the in silico validation of potential biomarkers of CCRT response, the transcriptomic data from a microarray dataset (GSE68204) of rectal cancer patients was downloaded from the National Center for Biotechnology Information-Gene Expression Omnibus (NCBI-GEO) database. This dataset was composed of 32 non-responders (NR) and 27 responders (R) rectal cancer patients. Notably, our in vitro studies, based on cell line models, confirmed the role of CHD4 in regulating radio-sensitivity in established radio-resistant clones and MSI clones.

## 2. Results

### 2.1. Identification of CHD4 as a Potential Biomarker Associated with Non-Responders to Pre-Operative CCRT of Rectal Cancer

**Figure 1:**
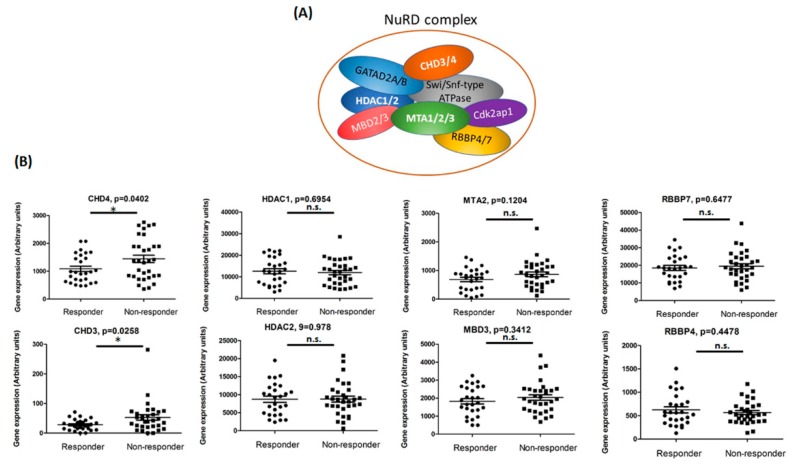
Gene expression analysis between responders and non-responders to concurrent chemoradiotherapy (CCRT). (**A**) Cartoon representation of the nucleosome remodeling and histone deacetylation (NuRD) complexes. (**B**) Correlation of gene expression between treatment responders (R) and non-responders (NR) to CCRT in rectal cancers patients. The RNA expression profiles from GSE68204 consisted of 32 NR and 27 R patients, as measured by tumor regression grade (TRG) (gene expression data were calculated using paired *t*-test). * indicated *p* < 0.05, and *n*.s. indicated no statistical significance.

We hypothesized that, differentially expressed genes between responders and non-responders to preoperative CCRT, may play crucial roles in therapeutic resistance. To identify these potential target genes, we analyzed a microarray dataset (GSE68204) from the NCBI-GEO database. The dataset comprised 59 clinical samples, of which 32 were NR and 27 were R to pre-operative CCRT. The NuRD complex is known to play a role in regulating DNA repair and gene expression. Thus, we focused on how the gene expression patterns of NuRD complex subunits (CHD4, CHD3, HDAC1, HDAC2, MTA2, MBD3, RBBP4, and RBBP7) vary between NR and R to pre-operative CCRT. We found significant upregulation of CHD3 and CHD4 in NR compared to R (*p* = 0.0258 and 0.0402, respectively) (Figure 1). This finding suggested that upregulation of CHD3 and CHD4 might be related to the differential therapeutic response to pre-operative CCRT among rectal cancers patients.

### 2.2. Relationship between CHD4 Expression and Clinico-Pathological Features

**Figure 2:**
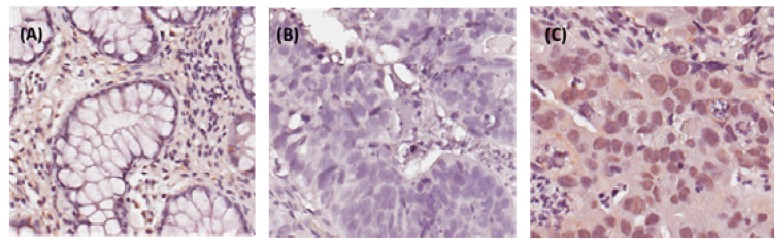
Immunohistochemical staining of CHD4 in representative human rectal tumor sections. (**A**) No expression in normal colonic mucosa. (**B**) Low CHD4 immuno-reactivity in tumors with high tumor regression grades following pre-operative chemo-radiation therapy. (**C**) High CHD4 immuno-reactivity in tumors with low tumor regression grades.

Our previous data demonstrated that CHD4 knockdown could sensitize cancer cells to DNA insult drugs in breast cancer and osteosarcoma [23,25]. Another previous study also indicated that, CHD4 is involved in oxidative DNA damage repair, for maintaining DNA hypermethylation-associated transcriptional silencing in CRC patients [26]. However, it is still unclear exactly how CHD4 may act as a radio-resistant regulator in rectal cancer patients. To address this question, we next performed immunohistochemical staining of 172 rectal cancer specimens to evaluate CHD4 expression in patients with rectal cancer, who are treated with CCRT. The immunostaining CHD4, in normal and tumor tissue, is illustrated in Figure 2. The relationships between CHD4 expression and clinico-pathological features are shown in Table 1. The high expression of CHD4 was significantly associated with pre-treatment (pre-Tx) tumor status (T3–T4 versus T1–T2; *p* < 0.001), pre-Tx lymph node metastasis (N1–2 versus N0; *p* < 0.001), post-treatment (post-Tx) tumor status (T3-T4 versus T1-T2; *p* < 0.001), post-Tx lymph node metastasis (N1–2 versus N0; *p* < 0.001), vascular invasion (*p* = 0.042), and tumor regression grade (*p* = 0.001).

### 2.3. High Expression of CHD4 Is Associated with Poor Prognosis in Rectal Cancers Patients

**Figure 3:**
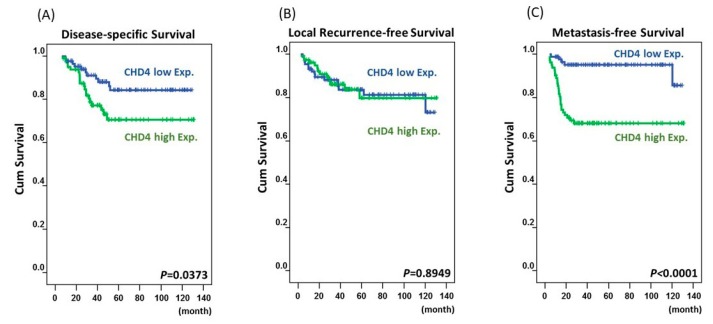
Kaplan-Meier survival curves of patients with varying CHD4 expression. High expression of CHD4 predicted inferior disease-specific survival (*p* = 0373) (**A**), but there was no significant difference in metastasis-free survival (*p* = 8949) (**B**). The CHD4 expression also demonstrated a significant prognostic impact on metastasis-free survival (*p* < 0001) (**C**).

Univariate analysis (Table 2 and Figure 3) revealed that disease-specific survival (DSS) was significantly associated with pre-Tx tumor status (*p* = 0.0484), pre-Tx lymph node metastasis (*p* = 0.0059), post-Tx tumor status (*p* = 0.0014), vascular invasion (*p* = 0.0123), tumor regression grade (*p* = 0.0037), and CHD4 expression (*p* = 0.0373). Local recurrence-free survival (LRFS) was significantly associated with pre-Tx nodal status (*p* = 0.0025), post-Tx tumor status (*p* = 0.0056), vascular invasion (*p* = 0.0023), perineural invasion (*p* = 0.0083), and tumor regression grade (*p* = 0.0021). Metastasis-free survival (MeFS) was significantly associated with post-Tx tumor status (*p* = 0.0123), tumor regression grade (*p* = 0.0008), and CHD4 expression (*p* < 0.0001). In the multivariate model of Cox proportional-hazards regression analysis (Table 3), we found that tumor regression grade was a significant prognostic factor for DSS (HR, 2.262; 95% CI, 1.1198–4.566; *p* = 0.023), LRFS (HR, 2.198; 95% CI, 1.002–4.831; *p* = 0.015), and MeFS (HR, 2.32; 95% CI, 1.063–4.292; *p* = 0.033). Interestingly, we found that CHD4 over-expression was an independent factor in the poor prognosis of MeFS (HR, 4.575; 95% CI, 1.717–12.192; *p* = 0.002), after adjusting for other clinical and pathological features, like tumor regression grade, vascular invasion, post-Tx tumor status, pre-Tx tumor status, pre-Tx nodal status, and perineural invasion.

### 2.4. CHD4 Regulates Resistance to Radiation in CRC Cells of Varying MSI Status

**Figure 4:**
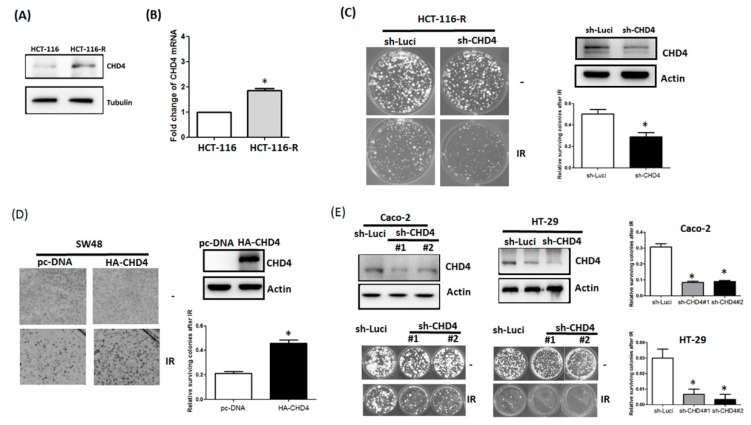
CHD4 regulates the radioresistance in CRC cells. (**A**). Protein expression of CHD4 in HCT-116 and radio-resistant HCT-116-R cells as determined by Western blotting. (**B**) Gene expression of CHD4 was determined by RT-qPCR in HCT-116 and radio-resistant HCT-116-R cells (paired *t*-test, *p* values). (**C**) Clonogenic assay with 1x10^3^ radio-resistant HCT-116-R cells and HCT-116-R-sh-CHD4 cells after exposure to ionizing radiation (IR) with the indicated dose for 2 weeks (paired *t*-test, *p* values). (**D**) Clonogenic assay with 1x103 cells of SW48 cells and SW48-CHD4 overexpressing cells after exposure to IR, with the indicated dose for 2 weeks. (paired *t*-test, *p* values). (**E**) Clonogenic assay, with 1 × 10^3^ cells of HT-29 cells and Cacco-2-CHD4 knockdown cells after exposure to IR, with the indicated dose for 2 weeks (One-way ANOVA, *p* values). * indicated *p* < 0.05.

To investigate the role of *CHD4* in the radio-resistance of CRC cells, we used both MSI (HCT-116 and SW48) and MSS (HT-29 and Caco-2) cell line models. To identify the effect of *CHD4* in predicting cell responses to ionizing radiation (IR) exposure, we established a radio-resistant cell line. The HCT-116 cell line, which is substantially sensitive to IR in vivo, was chosen as our in vitro cell-based model [25]. HCT-116-R cells survived exposure to 8 Gy IR from HCT-116 cells. As shown in Figure 4A, we found that the expression of CHD4 was markedly increased in radio-resistant HCT-116-R cells, which survived the exposure to IR. Consistent with the protein level, the mRNA levels of *CHD4* were also elevated in radio-resistant HCT-116-R cells after IR exposure (Figure 4B). Cells surviving from IR demonstrated high levels of CHD4. Knockdown of CHD4 in radio-resistant HCT-116-R cells led to enhanced cytotoxicity upon IR exposure, as compared to that in radio-resistant HCT-116-R (Figure 4C). Notably, the over-expression of CHD4 in SW48 cells led to pronounced radio-resistant phenotype, compared to parental SW48 cells (Figure 4D). We further validated the role of CHD4 in the radio-resistance of MSS cell line models (HT-29 and Caco-2). As shown in Figure 4E, the depletion of CHD4 in both HT-29 and Caco-2 cells increased their sensitivity to IR. Altogether, these results indicate that CHD4 regulates the resistance of CRC cells to IR.

## 3. Discussion

A high expression of CHD4 has been associated with poor prognosis in non-small-cell lung cancer and hepatocellular carcinoma [27,28]. Similarly, the upregulation of CHD4 has been reported in CRC patients with poor tumor differentiation, higher tumor nodal metastases status, stage, shorter overall survival, and higher recurrence [29]. However, there are no reports on the correlation between CHD4 upregulation and other pathological features, such as vascular invasion, perineural invasion, and most importantly, treatment response (tumor regression grade). In our study, the high expression of CHD4 was significantly associated with advanced tumor depth of invasion, nodal metastasis, and increased vascular invasion, all representing aggressive behavior. Noteworthy, after neoadjuvant CCRT, CHD4 expression was significantly correlated with advanced tumor and nodal status, post-CCRT and low tumor regression, which indicated that CHD4 is involved in therapeutic responses, which corresponded with clinical treatments.

The loss of CHD4 expression in BRCA-associated cancers, that are sensitive to DNA-damaging agents, such as cisplatin, leads to drug resistance [30]. In addition, CHD4 depletion affects ERBB2 and autophagy, and results in resistance to Trastuzumab [31]. Recent studies have reported that, the depletion of CHD4 sensitizes cancer cells to poly(ADP-ribose) polymerase (PARP) inhibitors and DNMT inhibitors, in both hematopoietic and solid tumors [24,28,32]. DNA methyltransferase (DNMT) inhibitors and histone deacetylase (HDAC) inhibitors increased the radio-sensitivity of head and neck squamous cell carcinoma [33]. Acombination of DNMT inhibitor and irradiation improved the radio-sensitivity of pancreatic cancer cells [34]. Thus, CHD4 may regulate cancer cell behavior through post-transcriptional modification, thereby regulating the sensitivity of cancer cells to various chemotherapeutic drugs. However, the impact of CHD4 in response to radiation therapy remains ambiguous. Our findings suggest that the expression of CHD4 is directly proportional to IR resistance, as a higher CHD4 expression was correlated with increased tolerance to IR. The deprivation of CHD4, in radio-resistant CRC cells, restored their sensitivity to IR.

Interestingly, the loss of CHD4 expression (defined as less than 30% of the neoplastic cells) correlates with CHD4 mutations, observed in 55.7% of the CRC patients. The mutations of the CHD family occur mainly in MSI-high (MSI-H), or in mismatch repair deficient (dMMR) cancers, as opposed to MSI-low (MSI-L)/MSS cancers [35]. Here, we use MSS cell lines to demonstrated that knockdown of CHD4 will turn MSS tumors into increased sensitivity to IR. It implies that the phenotype of CHD4 deficiency in MSS cells is similar to MSI-H tumor response to IR. Previous studies revealed that CHD4 suppressed p21 expression owing to its histone deacetylation activity, thus affecting drug response in Triple-negative breast cancer (TNBC) cells. Inhibition of CHD4 resulted in restoration of p21 expression and enhanced sensitivity to cisplatin and PARP inhibitors [36]. The radio-sensitivity observed in MSI-H CRC patients might be due to low CHD4 expression, which might result in increased acetylation of p21 promoter and concomitant p21 gene expression and contribute to its sensitivity to IR. On the contrary, HDAC inhibitors that suppress the NuRD complex activity could be an alternative therapeutic strategy for tumors with high CHD4 expression. In the study, we confirmed that microsatellite stable (MSS) CRC cells, with elevated CHD4 expression, were relatively resistant to IR, whereas the MSI-H CRC cells with low CHD4 expression were relatively radiosensitive. These in vitro findings suggest that, MSS CRC patients, who are more radioresistant, may require HDAC inhibitors to block the activity of the NuRD complex and restore p21 promoter acetylation, thereby resulting in enhanced p21 expression, and inducing sensitization to CCRT treatment. On the other hand, for radiosensitive MSI-H CRC patients with higher response to DNA-damaging agents or IR, CHD4 is a promising predictive biomarker and independent prognostic factor.

Nevertheless, our results showed discrepancies between DSS, LRFS, and MeFS. The associations with survival estimation were mainly obtained for DSS and MeFS. In our previous study, we found that CHD4 regulated the loss of E-cadherin and affected epithelial-mesenchymal transition (EMT), which promotes metastatic ability in breast cancer cell lines [37]. In CRCs, CHD4 knockdown activates TSGs and blunts proliferation, invasion, and metastases of tumor cells [38]. These results may explain the significance obtained for DSS and MeFS in our statistical analysis.

In conclusion, we demonstrated that the over-expression of CHD4 was negatively correlated with clinicopathological parameters, and poor responsiveness to neoadjuvant CCRT, in rectal cancer. In addition, a high expression of CHD4 was significantly associated with shorter disease-specific survival and metastasis-free survival in univariate analysis. Using multivariate analysis showed that, CHD4 is an independent biomarker to predict poor prognosis and low metastasis-free survival rates. Furthermore, we identified the important role of CHD4 in the radio-resistance of rectal cancer. Our in vitro experiments provide a new perspective on therapeutic strategies, combining radiotherapy with inhibitors of the NuRD complex, according to the CHD4 and MSI statuses of rectal cancer patients.

## 4. Materials and Methods

### 4.1. Microarray Data Analysis

In this study, we analyzed a microarray dataset (GSE68204) of rectal cancer patients, whereby a list was downloaded from the NCBI-GEO database. This dataset consisted of two groups of patients, with locally advanced rectal cancer, by 38 “exploration cohort”, and 21 “validation cohort”, respectively. A total of 32 non-responders (NR) and 27 responders (R) patients, as measured by tumor regression grade, treated with pre-operative chemoradiotherapy, were analyzed for gene expression experiments. The gene expression values were re-plotted, using GraphPad Prism 5.0 software (GraphPad, La Jolla, CA, USA).

### 4.2. Patients and Tissue Samples of Rectal Cancers

We collected the formalin-fixed paraffin-embedded (FFPE) specimens of 172 rectal cancer patients who underwent neoadjuvant CCRT, followed by radical proctectomy in Chi Mei Medical Center, between 1998 and 2004. This study was approved by the institutional review board of Chi Mei Medical Center (IRB10801001). In the initial state, we performed an endoscopic ultrasound (EUS) and abdominopelvic computed tomography (CT), in order to evaluate the clinical staging of rectal adenocarcinoma. We confirmed adenocarcinoma in all the patients by performing a colonendoscopic biopsy, and also confirmed that no distant metastasis existed via several examinations for staging. The clinical and pathological criteria were similar to those used in the previous study [25,37]. The pre-operative CCRT, included 5-fluorouracil-based chemotherapy and concomitant radiotherapy, with a total dose of 45 Gy in 25 fractions over a period of five weeks. Following surgical interventions, patients beyond T3 stage or nodal metastasis either, before, or after, CCRT received adjuvant chemotherapy. All patients were monitored regularly, according to previous studies [26]. The mean follow-up time in this cohort was 48.2 months (6.2–131.2).

### 4.3. Histopathological and Immunohistochemical Assessments

Two pathologists (CF Li and TJ Chen) evaluated the post-CCRT surgery specimens, according to the seventh edition of the cancer staining, developed by the American Joint Committee on Cancer (AJCC) [38]. We then combined this system of tumor regression grade (TRG) after neoadjuvant chemoradiotherapy, with the grading criteria reported by Dworak et al. [39], in order to obtain the following grades: ‘Grade 0′—no observed regression; ‘grade 1′—cancer cells with severe fibrosis and/or vasculopathy; ‘grade 2′—fibrosis with scattered cancer cells; ‘grade 3′—few scattered cancer cells on fibrosis background; ‘grade 4′—no visible cancer cells. The process of CHD4 immunohistochemical staining was similar to that previously reports [40,41]. In brief, FFPE tissues of a pre-treatment rectal tumor were de-paraffinized and rehydrated for CHD4 immunostaining. A 3% H_2_O_2_ treatment for 10 min was then applied to block endogenous peroxidase activity, and the tissues were washed with Tris-buffer saline for 15 min before incubation with anti-CHD4 monoclonal antibody (A301-081A, Bethyl Laboratories, Montgomery, TX, USA). Two pathologists (CF Li and TJ Chen) assessed CHD4 staining by the H-scoring method (H-score = Σ Pi (i + 1); ‘Pi’ symbolizes the percentage of stained tumor cells (0%–100%) and ‘i’ the ‘grade’ of staining intensity (0–3) [41,42], which was established in 1986 [43]. The CHD4 staining was then semi-quantitatively scored, incorporating both the distribution and the intensity of the specific staining. The assays were recorded as percentages of positively stained target cells in one of four intensity categories. The scores were based on the intensity of the signal (0, 1+, 2+, 3+) and the proportion on the positive cells (0 ≤ 10%, 1 = 10–25%, 2 = 25–50%, 3 ≥ 50%). The staining index was calculated as the product of signal intensity and proportion of positive cells. All staining results were reviewed and scored independently by two pathologists. High expression of CHD4 in tumors was defined as greater than the median expression in all samples. Immunohistochemically, CHD4 has been localized to the nucleus of tumor cells [29].

### 4.4. Statistical Analysis

The significance of the microarray gene expression analysis was determined using an unpaired *t*-test to analyze two groups or one-way ANOVA, with Tukey’s post hoc test, and to make comparisons between multiple groups using GraphPad Prism 5.0 software.

A statistical analysis was performed by using the SPSS 14 software package. We used Chi-square test to analyze the association of CHD4 expression with clinical and pathological features of rectal cancer patients. We also calculated the time interval between the date of surgical intervention and the date of endpoint events including disease-specific survival, local recurrence-free survival, and metastasis-free survival (MeFS), as previously described in [44]. The Kaplan-Meier method was used to plot the survival curves and log-rank tests were used to evaluate the significant difference in prognosis between different subgroups. Cox regression analysis was applied to assess the prognostic significance in univariate and multivariate models. For all two-tailed analyses, *p* values < 0.05 were considered significant.

### 4.5. Cell Lines, Reagents and Plasmids

Two MSI-high (HCT-116 and SW48 cells) cell lines and two micro-satellite stable (Caco-2 and HT-29) cell lines were cultured in Dulbecco’s modified Eagle’s medium (DMEM), supplemented with 10% fetal bovine serum (FBS). HCT-116-R cells were survived exposure to 8 Gy IR from HCT-116 cells. Plasmids containing shRNA sequences against CHD4 were obtained from the National RNAi Core Facility (Academia Sinica, Taiwan). Sequences of shCHD4 was: 5′-CCTTACTAGAATTGGTGTTAT-3′; and control sh-luciferase: 5′-CTTCGAAATGTCCGTTCGGTT-3′. Antibodies against CHD4 (GTX124186) and Actin (GTX112794) were purchased from Genetex (SanAntonio, TX, USA).

### 4.6. Quantitative Reverse-Transcription PCR (RT-qPCR)

The total RNA was extracted, using an RNeasy mini kit (Qiagen, Valencia, CA, USA), according to the manufacturer’s instructions. Equal amounts of RNA were converted to first-strand cDNA, using the RT2 first strand kit (Qiagen, Valencia, CA, USA), as previously described in [45]. qRT-PCR was performed using SYBR Green master mix in Real-Time PCR System (Applied Biosystems, Foster City, CA, USA). The primer sequences of CHD4 are 5′-GGTTTTGGTTCCAAGCGTAA-3′ (forward) and 5′-CTCCTCCTCGCCTTTCTTTT-3′ (reverse).

### 4.7. Immunoblotting and Immunohistochemistry

Protein extraction and immunoblotting were performed, as previously described [43]. Briefly, protein lysis buffer (M-PERTM mammalian protein extraction buffer, Thermo Fisher Scientific, Rockford, IL, USA) was used for cell lysis, followed by centrifugation at 13,000 rpm, after which the supernatant, containing protein, was collected. Proteins were run on an SDS-polyacrylamide gel and then transferred to a nitrocellulose membrane. Immunoblotting was performed, using anti-CHD4 and anti-Actin antibodies, and detected with secondary antibodies conjugated to HRP. For immunohistochemistry, paraffin tissue samples were sectioned at 4 µm thickness, approximately, de-paraffinized, rehydrated, and autoclaved to induce antigen retrieval with citrate buffer (10 mM citric acid, 0.05% Tween 20, pH 6.0). Then, tissue sections were incubated with CHD4 primary antibodies (1:1000; GeneTex Inc., Irvine, CA, USA) for 1 hour, and finally analyzed, using the detection kit (DAKO, Carpinteria, CA, USA).

### 4.8. Colony Formation Assays

In brief, 5 × 10^2^ HCT-116 cells were transfected with shRNA and/or control plasmid, and seeded in 6-well adherent plates (Corning, Tewksbury, MA, USA). Following irradiation, the growth medium was replenished every 3 days. The colonies, which were formed were imaged at 14 days post-treatment, to detect colony size. The colonies were defined as groups ≥ 50 cells.

## Figures and Tables

**Table 1:** Associations and comparisons between CHD4 expression and clinicopathological factors in 172 rectal cancer patients who were receiving neoadjuvant CCRT. High expression of CHD4 was significantly associated with pre-Tx tumor status (*p* < 0.001), pre-Tx lymph node metastasis (*p* < 0.001), post-Tx tumor status (*p* < 0.001), post-Tx lymph node metastasis (*p* < 0.001), vascular invasion (*p* = 0.042), and tumor regression grade (*p* = 0.001).

Parameter		No.	CHD4 Expression		*p*-Value
			Low Exp.	High Exp.	
Gender	Male	108	60	48	0.194
	Female	64	29	35	
Age	<70	106	61	45	0.054
	≥70	66	28	38	
Pre-Tx tumor status (Pre-T)	T1–T2	81	55	26	<0.001 *
	T3–T4	91	34	57	
Pre-Tx nodal status (Pre-N)	N0	125	77	48	<0.001 *
	N1–N2	47	12	35	
Post-Tx tumor status (Post-T)	T1–T2	86	57	29	<0.001 *
	T3–T4	86	32	54	
Post-Tx nodal status (Post-N)	N0	123	76	47	<0.001 *
	N1–N2	49	13	36	
Vascular invasion	Absent	157	85	72	0.042 *
	Present	15	4	11	
Perineural invasion	Absent	167	88	79	0.149
	Present	5	1	4	
Tumor regression grade	Grade 0–1	37	11	26	0.001 *
	Grade 2–3	118	64	54	
	Grade 4	17	14	3	

*, statistically significant.

**Table 2:** Univariate log-rank analysis for important clinicopathological variables and CHD4 expression. In the multivariate regression analysis, CHD4 over-expression was an independent factor of poor prognosis for MeFS (*p* = 0.002) after adjustment.

Parameter		No. of Case	DSS		LRFS		MeFS	
			No. of Event	*p*-Value	No. of Event	*p*-Value	No. of Event	*p*-Value
Gender	Male	108	20	0.6027	5	0.3096	14	0.1047
	Female	64	11		17		15	
Age	<70	106	19	0.7158	14	0.9630	18	0.9520
	≥70	66	12		8		11	
Pre-Tx tumor status (Pre-T)	T1–T2	81	10	0.0484 *	7	0.0836	10	0.1288
	T3–T4	91	21		15		19	
Pre-Tx nodal status (Pre-N)	N0	125	19	0.0059 *	12	0.0025 *	18	0.0866
	N1–N2	47	21		10		11	
Post-Tx tumor status (Post-T)	T1–T2	86	7	0.0014 *	5	0.0056 *	8	0.0123 *
	T3–T4	86	24		17		21	
Post-Tx nodal status (Post-N)	N0	123	21	0.4654	15	0.6267	20	0.8403
	N1–N2	49	10		7		9	
Vascular invasion	Absent	157	25	0.0123 *	17	0.0023 *	26	0.7236
	Present	15	6		5		3	
Perineural invasion	Absent	167	29	0.0994	20	0.0083 *	28	0.8157
	Present	5	2		2		1	
Tumor regression grade	Grade 0–1	37	13	0.0037 *	10	0.0021 *	14	0.0008 *
	Grade 2–3	118	17		12		14	
	Grade 4	17	1		0		1	
Down stage after CCRT	Non-Significant	150	29	0.2348	20	0.5234	28	0.1291
	Significant (≥2)	22	2		2		1	
CHD4 expression	Low Exp.	89	11	0.0373 *	15	0.8949	5	<0.0001 *
	High Exp.	83	20		12		26	

*, statistically significant.

**Table 3:** Multivariate analysis.

Parameter	DSS			LRFS			MeFS		
	H.R	95% CI	*p*-Value	H.R	95% CI	*p*-Value	H.R	95% CI	*p*-Value
Tumor regression grade	2.262	1.1198–4.566	0.023 *	2.198	1.002–4.831	0.015 *	2.32	1.063–4.292	0.033 *
CHD4 expression	1.181	0.519–2.686	0.692	-	-	-	4.575	1.717–12.192	0.002*
Vascular invasion	2.082	0.771–5.622	0.148	2.510	0.902–6.985	0.078	-	-	-
Post-Tx tumor status (Post-T)	2.447	0.992–6.034	0.052	2.041	0.825–5.051	0.123	1.736	0.751–4.012	0.197
Pre-Tx nodal status (Pre-N)	1.286	0.538–3.070	0.571	1.993	0.833–4.770	0.121	-	-	-
Pre-Tx tumor status (Pre-T)	1.283	0.532–3.096	0.579	-	-	-	-	-	-
Perineural invasion	-	-	-	1.122	0.231–5.447	0.887	-	-	-

H.R., hazard ratio *, statistically significant.
